# Detection and evolutionary dynamics of somatic FAS variants in autoimmune lymphoproliferative syndrome: Diagnostic implications

**DOI:** 10.3389/fimmu.2022.1014984

**Published:** 2022-11-18

**Authors:** Laura Batlle-Masó, Marina Garcia-Prat, Alba Parra-Martínez, Clara Franco-Jarava, Aina Aguiló-Cucurull, Pablo Velasco, María Antolín, Jacques G. Rivière, Andrea Martín-Nalda, Pere Soler-Palacín, Mónica Martínez-Gallo, Roger Colobran

**Affiliations:** ^1^ Infection in Immunocompromised Pediatric Patients Research Group, Vall d’Hebron Research Institute (VHIR), Barcelona, Spain; ^2^ Pediatric Infectious Diseases and Immunodeficiencies Unit, Vall d’Hebron University Hospital (HUVH), Barcelona, Spain; ^3^ Jeffrey Modell Diagnostic and Research Center for Primary Immunodeficiencies, Barcelona, Spain; ^4^ Translational Immunology Group, Vall d’Hebron Research Institute (VHIR), Barcelona, Spain; ^5^ Immunology Division, Vall d’Hebron University Hospital (HUVH), Barcelona, Spain; ^6^ Pediatric Oncology and Hematology Department, Vall d’Hebron University Hospital (HUVH), Barcelona, Spain; ^7^ Department of Clinical and Molecular Genetics, Vall d’Hebron University Hospital (HUVH), Barcelona, Spain; ^8^ Department of Cell Biology, Autonomous University of Barcelona (UAB), Physiology and Immunology, Bellaterra, Spain

**Keywords:** primary immunodeficiencies, inborn errors of immunity, autoimmune lymphoproliferative syndrome, FAS, somatic mutation, genetics, next-generation sequencing

## Abstract

Autoimmune lymphoproliferative syndrome (ALPS) is a rare primary immune disorder characterized by impaired apoptotic homeostasis. The clinical characteristics include lymphoproliferation, autoimmunity (mainly cytopenia), and an increased risk of lymphoma. A distinctive biological feature is accumulation (>2.5%) of an abnormal cell subset composed of TCRαβ^+^ CD4^-^CD8^-^ T cells (DNTs). The most common genetic causes of ALPS are monoallelic pathogenic variants in the *FAS* gene followed by somatic *FAS* variants, mainly restricted to DNTs. Identification of somatic *FAS* variants has been typically addressed by Sanger sequencing in isolated DNTs. However, this approach can be costly and technically challenging, and may not be successful in patients with normal DNT counts receiving immunosuppressive treatment. In this study, we identified a novel somatic mutation in *FAS* (c.718_719insGTCG) by Sanger sequencing on purified CD3^+^ cells. We then followed the evolutionary dynamics of the variant along time with an NGS-based approach involving deep amplicon sequencing (DAS) at high coverage (20,000-30,000x). Over five years of clinical follow-up, we obtained six blood samples for molecular study from the pre-treatment (DNTs>7%) and treatment (DNTs<2%) periods. DAS enabled detection of the somatic variant in all samples, even the one obtained after five years of immunosuppressive treatment (DNTs: 0.89%). The variant allele frequency (VAF) range was 4%-5% in pre-treatment samples and <1.5% in treatment samples, and there was a strong positive correlation between DNT counts and VAF (Pearson’s R: 0.98, p=0.0003). We then explored whether the same approach could be used in a discovery setting. In the last follow-up sample (DNT: 0.89%) we performed somatic variant calling on the *FAS* exon 9 DAS data from whole blood and purified CD3^+^ cells using VarScan 2. The c.718_719insGTCG variant was identified in both samples and showed the highest VAF (0.67% blood, 1.58% CD3^+^ cells) among >400 variants called. In summary, our study illustrates the evolutionary dynamics of a somatic *FAS* mutation before and during immunosuppressive treatment. The results show that pathogenic somatic *FAS* variants can be identified with the use of DAS in whole blood of ALPS patients regardless of their DNT counts.

## Introduction

Autoimmune lymphoproliferative syndrome (ALPS, OMIM #601859) is characterized by a lack of control over lymphocyte apoptosis. This results in an elevated number of lymphocytes, which can accumulate and lead to enlarged lymph nodes, liver, and spleen. The main clinical features in ALPS are lymphadenopathy, hepatomegaly, splenomegaly, and autoimmune cytopenia. There is also an increased risk for developing severe health conditions such as autoimmune diseases and hematological malignancies (Hodgkin and non-Hodgkin lymphoma) (1).

The diagnosis of ALPS is based on two major criteria: elevated (>2.5%) CD3^+^ TCRαβ^+^ CD4^-^CD8^-^ T cells (hereafter referred to as double-negative T cells, [DNTs]), and chronic nonmalignant/noninfectious lymphoproliferation. The accessory criteria include defective lymphocyte apoptosis, presence of pathogenic genetic variants, elevated levels of soluble molecules such as FAS ligand, IL10 and vitamin B12, typical immunohistological findings, autoimmune cytopenia, and a positive family history (2). Immunosuppressive drugs, such as steroids, mycophenolate and the mTOR inhibitor rapamycin (sirolimus), are good treatment options for ALPS patients (3), as they effectively reduce cytopenia and lymphoproliferation. However, patients should be monitored to control drug-related toxicity. Hematopoietic stem cell transplantation (HSCT) has been successful for curing severe ALPS, but it is a complex procedure and the risk/benefit balance must be carefully evaluated in each patient (4).

In most cases, the molecular origin of ALPS is associated with pathogenic germline variants in the *FAS* gene, which encodes the FAS cell surface death receptor (5, 6). Most of these variants are monoallelic (autosomal dominant), affect the intracellular death domain (DD) region, and exert a dominant-negative effect related to the trimeric nature of the FAS protein (7). Heterozygous loss-of-function variants leading to *FAS* haploinsufficiency without a dominant negative effect are a less frequent cause. These variants are usually located in the FAS extracellular region and are associated with milder clinical manifestations and lower penetrance (8). In addition, biallelic germline variants in *FAS* have been reported in severe early-onset ALPS patients (6, 9). In these cases, heterozygous carriers were asymptomatic, indicating that some pathogenic *FAS* variants behave as recessive alleles whereas others are dominant (1).

Pathogenic germline variants in other genes affecting the FAS-related apoptosis pathway have been reported as a cause of ALPS in a minor fraction of patients: *FASLG* (FAS ligand) (10), *FADD* (FAS-associated death domain protein) (11), *CASP8* (caspase 8) (12), and *CASP10* (caspase 10) (13). However, the role of *CASP10* variants as a causative versus predisposing factor in ALPS is still controversial (14).

After germline *FAS* mutations, heterozygous somatic *FAS* mutations are the second most common genetic etiology of ALPS, accounting for up to 15% of patients (15). Several features of somatic *FAS* variants have been reported to date: 1) They appear in hematopoietic progenitors and are predominantly detected in DNTs, although they are also present in smaller proportions in other lymphoid and myeloid lineages (15); 2) They are mainly located in exons 7, 8 and 9 of the *FAS* gene, encoding the intracellular domain (16); and 3) They cause clinical phenotypes indistinguishable from those caused by germline *FAS* variants (17).

To further complicate the molecular basis of ALPS, other inheritance patterns have been described, including the double-hit hypothesis involving “second-hit” variants in the *FAS* gene itself or in other disease-modifying genes (18).

New sequencing technologies, such as deep amplicon sequencing, have enabled better and more comprehensive detection of true pathogenic somatic variants in next-generation sequencing (NGS) data (19). Optimized callers for somatic variation are being developed and improved (20), and although somatic variants have been traditionally investigated in the context of cancer, their role in immune-mediated disorders is increasingly recognized (18, 19, 21, 22).

Here, we present an analysis of data from a pediatric patient in whom a novel pathogenic somatic variant was identified and studied over five years. We evaluated the dynamics of the variant, the DNT population, and the patient’s clinical course, focusing on future application of this strategy in a discovery setting for patients with no available pretreatment samples. In addition, we performed a literature review to gather all pathogenic somatic variants associated with ALPS to facilitate identification of future novel variants. Our results provide additional information on the genetic bases of the disease and clues to improve diagnostic rates and validate the effectiveness of treatment at the molecular level.

## Materials and methods

### Patient and samples

The patient included in this study was attended during a five-year period (2017–2021) at Hospital Universitari Vall d’Hebron (HUVH) (Barcelona, Catalonia, Spain). Written informed consent for the studies reported here and for publication was obtained from the patient’s legal representatives, according to the procedures of the Ethics Review Board of Hospital Universitari Vall d’Hebron [code: PR(AG)69/2016].

### Evaluation of DNTs by flow cytometry

Whole blood collected in EDTA was stained with the following antibodies—TCRαβ-FITC, CD4-PE, CD3-APC, CD8-PerCP-Cy5—and analyzed with a FACSCanto II flow cytometer (Becton Dickinson). Data were processed with Kaluza analysis software (Beckman Coulter) using the following gating strategy: FSC vs SSC (lymphocyte selection), CD3^+^ T cell selection, TCRαβ selection, and finally CD4 and CD8 dot plot selecting DNT lymphocytes. The double-negative population was reported over the CD3^+^ population.

### CD3+ cell enrichment

Human CD3^+^ cells were isolated from 8 mL of heparinized whole blood by negative selection using the EasySep Human T Cell Isolation Kit (#17951, Stemcell Technologies, Vancouver, BC, Canada) in 2017, and the RosetteSep Human T Cell Enrichment Cocktail Kit (#15021, Stemcell Technologies) in 2021. Purification was confirmed by flow cytometry, obtaining >95% purity in both cases. CD3^+^ pellets were stored at -80°C until use.

### Genetic analysis by Sanger sequencing

All exons and flanking regions of the *FAS*, *CASP8*, and *CASP10* genes were sequenced by Sanger method (**Supplementary Table 1**). Genomic DNA was extracted from EDTA-containing whole-blood samples using the QIAamp DNA blood mini kit (Qiagen; Hilden, Germany) according to the manufacturer’s instructions. A polymerase chain reaction (PCR) technique was carried out to amplify exons and flanking regions. A 40-ng amount of genomic DNA was used in each PCR reaction. *FAS* exon 9 was amplified using the following oligonucleotides: forward 5’-TCTTGTTTCTGTATTCCCCTAGTCAGC-3’, reverse 5’-AAACCAAGCAGTATTTACAGCCAGC-3’ (amplimer size: 570bp). An internal oligonucleotide was used for sequencing: 5’-AGACCTTTAGGACTTAGCTAT-3’. PCR conditions (35 cycles) were as follows: 95°C for 30 s, 62°C for 30 s and 72°C for 40 s. Purified PCR products were sequenced on an ABI 3100 DNA sequencer using the BigDye Terminator sequencing kit, 3.1 (Applied Biosystems; Foster, VA, USA).

### Genetic analysis by NGS-based deep amplicon sequencing (DAS)

*FAS* exon 9 amplimers containing the whole exon and flanking regions were PCR-generated using 40-ng of DNA from whole blood or CD3^+^ cells. Two different sets of primers were used to ensure unbiased amplification, obtaining equivalent DAS results. Primer pair 1 was the same as that used for Sanger sequencing and primer pair 2 was the following: forward 5’-TGGGTATATGGCAGGATTTGGA-3’, reverse 5’-CCTTGGAGGCAGAATCATGAG-3’ (amplimer size: 695bp). PCR conditions for primer pair 2 (35 cycles) were 95°C for 30 s, 58°C for 30 s and 72°C for 45 s. PCR products were quantified with a Qubit 2.0 Fluorometer (Thermo Fisher Scientific, MA, USA). A 300- to 900-ng amount of DNA was fragmented using NEBNext dsDNA Fragmentase (New England Biolabs, Ipswich, MA, USA) to obtain fragments of approximately 200bp in size. End repair, ligation of the adapter for Illumina, and sample indexing by a small amplification were then carried out using the NEBNext Ultra DNA library prep kit for Illumina and the NEBNext MultiplexOligos for Illumina Dual Index Primers Set 1 (New England Biolabs, Ipswich, MA, USA). All necessary purifications and size selections to recover only the fragments of interest were done using AMPure XP beads (Beckman Coulter). Libraries were quality-evaluated using QIAxcel (Qiagen) and quantified with a Qubit 2.0 Fluorometer. The DNA sample libraries were then mixed in equimolecular amounts and sequenced in a MiSeq instrument (Illumina, San Diego, CA, USA) using the 500-cycle MiSeq reagent kit v2 with a paired-end run of 2×250-bp reads. All procedures were performed according to the manufacturer’s instructions.

### Bioinformatic analysis

Results were aligned to the reference genome, hg38, using BWA (https://github.com/lh3/bwa). Duplicate reads were marked using Picard MarkDuplicates (version 2.18.6, https://github.com/broadinstitute/picard). Next, GATK 4.1.8.1(https://github.com/broadinstitute/gatk) was used to calibrate and calculate BQSR scores (BaseRecalibrator, ApplyBQSR). Filtering was then done using SAMtools (https://github.com/samtools/samtools) to discard low-quality reads (mapping quality < 30, samtools view -bSq 30) and secondary alignments (samtools view -F 256). Each allele was counted using the Pysam Python package, version 0.15.2 (https://github.com/pysam-developers/pysam), using the fetch function as indicated by the package documentation (https://pysam.readthedocs.io/en/latest/api.html). Next we manually validated using the integrative genomics viewer (IGV) software (https://software.broadinstitute.org/software/igv/). Additional somatic variant-calling was performed with VarScan 2, version 2.4.340 (https://sourceforge.net/projects/varscan/files/) to compare the causal genetic variant with other hits found by the software; off-target reads and those of low quality (less than 500 total counts) were excluded.

### Literature review

A comprehensive literature search in PubMed, Google Scholar, SNPdb, ClinVar, HGMD, and Ensembl was conducted to gather all *FAS* gene somatic variants described in ALPS patients. To retrieve the maximum number of peer-reviewed papers, we combined (among others) the following keywords: ALPS, autoimmune lymphoproliferative syndrome, somatic, FAS, post-zygotic, TNFRSF6, mutation, variant, and genetic variant. Each article was manually curated, and additional information on each variant was annotated to the final table using UniProt, gnomAD, and Varsome. ProteinPaint (23), was used to create a base figure which was then adapted to our final version.

## Results

### Discovery of a novel pathogenic somatic variant underlying ALPS in a pediatric patient

**Figure 1:**
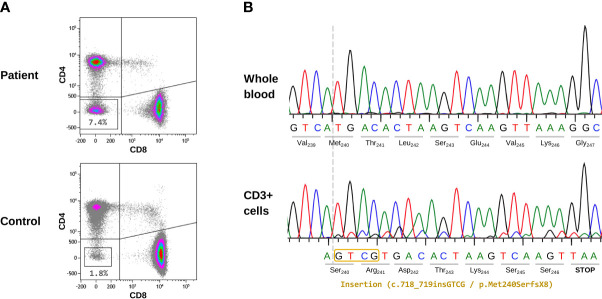
Cellular and molecular characterization of the patient’s samples. **(A)** Evaluation of TCRαβ^+^ CD4^-^CD8^-^ T cells (DNTs) by flow cytometry. The index patient and a representative healthy donor are shown. Gating strategy: on CD3^+^ T cells, TCRαβ positive cells were first selected; next, CD4 and CD8 dot-plot analysis was performed. The lower-left quadrant contains DNT data (percentage of DNTs) is indicated. **(B)** Sanger sequencing results from the initial sample (2017). The upper panel corresponds to DNA extracted from whole blood where no somatic variant was detected. The lower panel shows the CD3^+^ enriched sample, where a double peak pattern appeared, indicating the presence of a 4-bp insertion (c.718_719insGTCG).

The patient under study was an infant boy who presented with recurrent nonspecific fever, splenomegaly, diarrhea, and upper respiratory tract infections at 11 months of age (**Table 1**). He was born in Bolivia to non-consanguineous parents, and there was no family history of immune disorders. Laboratory analysis showed thrombocytopenia (platelet count: 67x10^9^/L; reference range: 150-500x10^9^/L), anemia (hemoglobin: 7.9 g/dL; reference range: 11.5-13.5 g/dL), monocytosis (absolute monocyte numbers: 1.6x10^9^/L; reference range: 0.1-0.7x10^9^/L), hypergammaglobulinemia (IgG: 1882 mg/dL; reference range: 196-1046 mg/dL), and elevated IL10 (132 pg/mL; reference range 0-7.8 pg/mL) and vitamin B12 (>2000 pg/mL; reference range: 211-911 pg/mL) levels. DNTs were evaluated to investigate suspected ALPS, and the value obtained was clearly elevated (7.4%; reference range <2.5%) (**Figure 1A**).

**Table 1:** Information on the clinical diagnosis, laboratory tests, and treatment of the index patient starting from the initial visit (May 2017) to the latest follow up (March 2021).

	2017 (May)	2017 (August)	2017 (November)	2018 (May)	2018 (December)	2021 (March)
**Age**	12 months	15 months	17 months	2 years	2.5 years	5 years
**Clinical data**	Erythema, cough, nonspecific fever	Respiratory infection, splenomegaly, fever	Respiratory infection, mild splenomegaly, without fever	Clinically asymptomatic	Clinically asymptomatic	Clinically asymptomatic
**Laboratory data**	DNTs elevated (7.4%)	DNTs elevated (7.8%)	DNTs within normal (1.9%)	DNTs within normal (1.4%)	DNTs within normal (1.76%)	DNTs within normal (0.89%)
**Treatment**	None	None	Sirolimus^a^	Sirolimus	Sirolimus	Sirolimus

At each time point, a blood sample was obtained for molecular analysis. DNTs, TCRαβ^+^ CD4^-^CD8^-^ T cells

a Sirolimus treatment was started at 15 months, just after the second follow up (August 2017).

Direct sequencing (Sanger method) of the *FAS*, *CASP8*, and *CASP10* genes ruled out pathogenic germline variants in these genes. As somatic mutations in *FAS* (mainly limited to DNTs) are the second most common genetic cause of ALPS, we addressed this possibility by purifying and sequencing (Sanger method) CD3^+^ cells from whole blood. Although the gold standard is to isolate DNTs by cell sorting, we performed a more accessible method: CD3^+^ cell purification by magnetic beads. In our sample, DNTs represented 7.4% of all CD3^+^ cells; therefore, somatic variants were enriched in this cell population compared with that of whole blood. We observed a small double peak pattern in exon 9 of *FAS*, consisting of a 4-bp insertion (c.718_719insGTCG), which could have corresponded to a somatic variant. Sequencing was repeated twice with the same results, and the small double peak pattern was not visible in DNA from whole blood (**Figure 1B**). The size of the peaks corresponding to the somatic variant was concordant with the 7.4% of DNTs where the variant was expected to be present. This novel insertion was predicted to have a frameshift effect and to cause a premature STOP codon (p.Met240SerfsX8). It is located at an evolutionarily highly conserved position (CADD v1.6 = 23) in the intracellular *FAS* region where most somatic variants have been reported (16). The variant was not found in the main population databases (ExAC, gnomAD) and has not been described in the literature. We classified the variant as pathogenic, reported it to ClinVar (accession number: SCV002568438), and considered it to be the genetic cause of the patient’s clinical phenotype.

### Evolutionary dynamics of the somatic *FAS* variant and DNTs along time

**Figure 2:**
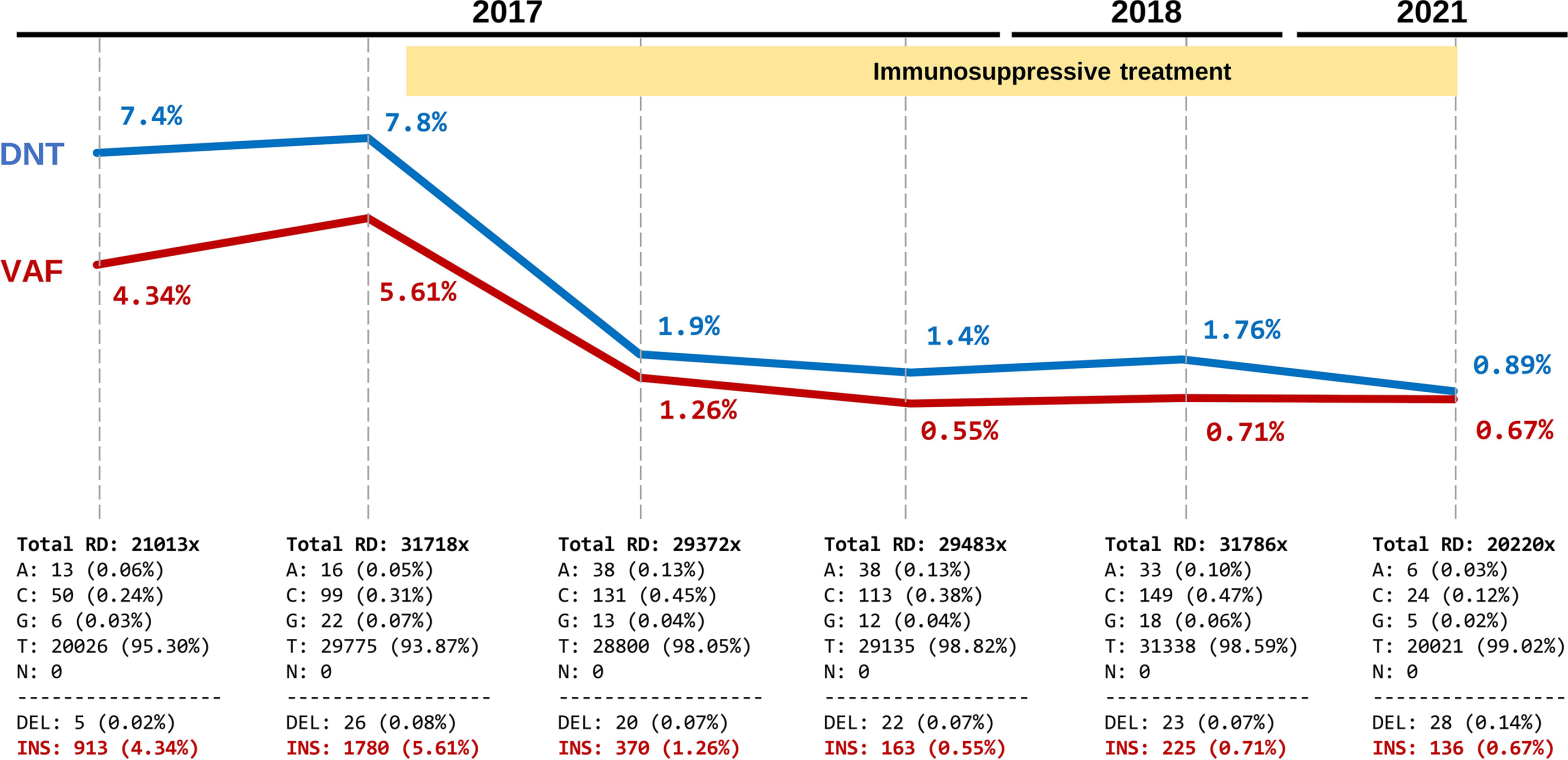
Evolutionary dynamics of the somatic *FAS* variant and correlation with DNT percentages during a five-year follow up. Each time point shows the TCRαβ^+^ CD4^-^CD8^-^ T cells (DNTs) (in blue) and variant allele frequency (VAF) of the pathogenic somatic *FAS* variant (c.718_719insGTCG) (in red). The yellow shading indicates the sirolimus treatment period. The bottom panels show the absolute values and percentage of read counts supporting each allele in the position of the variant c.718_719insGTCG (values were extracted from IGV data after quality filtering). Total RD, Total read depth.

At the age of 15 months, the patient was started on immunosuppressive treatment with sirolimus (2 mg/m^2^ per day), which significantly improved the clinical symptoms and markedly reduced the number of DNTs. During the next five years, the patient remained asymptomatic and sirolimus dose was periodically adjusted according to weight. During this clinical follow-up (2017–2021), six blood samples were obtained and stored for molecular studies: two in the pre-treatment period and four while receiving treatment (**Table 1**). DNT counts were assessed in each sample. Using NGS-based deep amplicon sequencing (DAS) at high coverage (20,000-30,000x), we aimed to detect and track the c.718_719insGTCG somatic variant in all samples (whole blood). First, we confirmed the presence of the variant in the initial sample at a variant allele frequency (VAF) of 4.3% (**Figure 2**). Second, we detected the somatic variant in all samples, even in the one obtained after five years of treatment. At that time, DNT counts were within the reference range (0.89%) and the variant was still detectable, although at a low VAF (0.67%). Third, we observed a strong positive correlation between DNT counts and the VAF (Pearson’s R: 0.98, p=0.0003). These results confirmed that the somatic variant was mainly restricted to DNTs and that cells derived from the mutated clone were present in peripheral blood at very low but detectable levels even after years of immunosuppressive treatment.

### Detection of somatic *FAS* variants under immunosuppressive treatment: Diagnostic implications

Detection of pathogenic somatic *FAS* variants in ALPS can be relatively straightforward when the patient has elevated DNTs that can be isolated by cell sorting and Sanger sequenced. Nevertheless, sometimes patients are already under immunosuppressive treatment when the search for somatic *FAS* variants is considered. In this situation, the number of DNTs is likely within the reference range and the process of isolation by cell sorting and Sanger sequencing may be unsuccessful.

**Figure 3:**
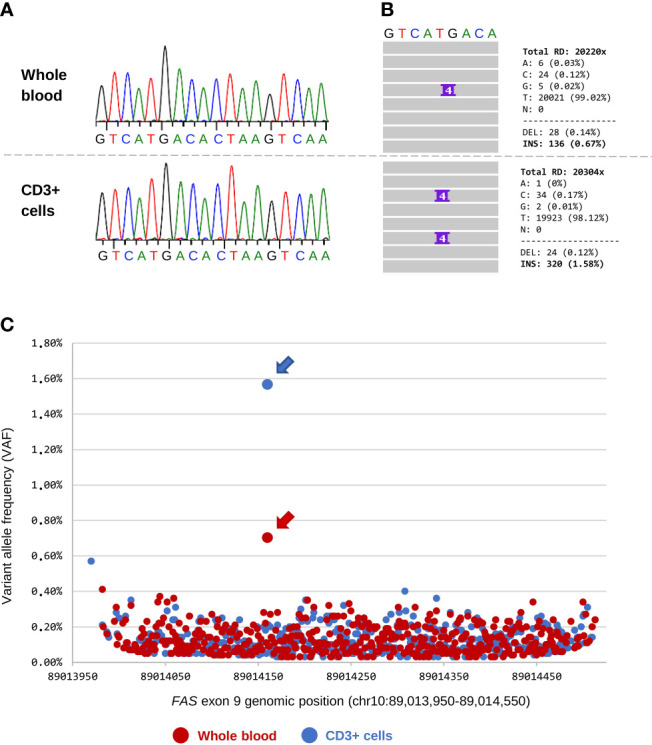
Detection of the somatic *FAS* variant in blood and CD3^+^ cells. **(A)** Sanger sequencing of whole blood and CD3^+^ cells (sample obtained in 2021). DNT count at that time accounted for 0.89% of the total CD3^+^ lymphocytes. There is no evidence of the somatic variant in chromatograms of the two samples. **(B)** Details of the deep amplicon sequencing results for the two samples. IGV screenshot and total read counts are shown. **(C)** Chromosome position and variant allele frequency (VAF) of all presumed somatic variants called by VarScan 2 in exon 9 of the *FAS* gene (hg38). Each dot is a variant: 470 variants were obtained from whole blood (dots in red) and 450 from CD3^+^ cells (dots in blue). The pathogenic somatic variant (chr10:89,014,160; c.718_719insGTCG) is indicated with an arrow.

Over the patient’s five-year follow-up, the pathogenic somatic variant was detected by DAS in all samples regardless of the DNT count. However, we had the advantage of knowing *a priori* the candidate variant. We then aimed to explore whether the same approach could be used in a discovery setting. For that purpose, we focused on the last follow-up, when the patient had been receiving immunosuppressive treatment for five years and DNTs were present at only 0.89% (**Table 1**, 2021). Whole blood and purified CD3^+^ cells were obtained using a simple method based on standard density gradient centrifugation with RosetteSep. We sequenced the entire exon 9 of *FAS* from blood and CD3^+^ cells by Sanger and DAS (coverage 20,000x). Sanger sequencing showed no trace of the somatic variant in either whole blood or CD3^+^ cells (**Figure 3A**). This was expected because the percentage of DNTs in the two samples was below the sensitivity of the technique. In contrast, with the use of DAS, the somatic variant was detected in both blood and CD3^+^ cells (**Figure 3B**).

As was mentioned above, we were aware of the candidate and this greatly facilitated analysis of the DAS data. However, we wanted to go one step further and determine whether the pathogenic somatic variant could have been identified with no *a priori* knowledge. To that end, we performed somatic variant calling on the raw BAM files from the DAS analysis using VarScan 2. The software called 470 candidate variants from whole blood and 450 from CD3^+^ cells (**Figure 3C**). It is known that only a small percentage of somatic calls obtained with this type of software are true somatic variants (24); therefore, we used the VAF and p-values to differentiate between background noise and true variants. The background noise was similar in the two samples, with most variants detected showing a VAF <0.4% (**Figure 3C**) and a p-value <1x10^-4^ (**Supplementary Figure 1** **).** The c.718_719insGTCG variant was identified in both samples and showed the highest VAF (0.67% in whole blood and 1.58% in CD3^+^ cells) and lowest p-value (1x10^-7^ in whole blood and 1x10^-20^ in CD3^+^ cells) of all variants identified (**Figure 3C** and **Supplementary Figure 1**). Remarkably, the simple CD3 enrichment step clearly improved the process of detecting true somatic variants, increasing the VAF and p-value by more than 2-fold each.

### Review of somatic *FAS* variants reported in ALPS patients

**Figure 4:**
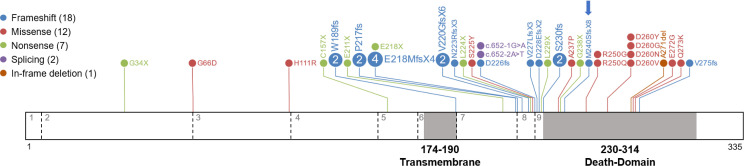
Somatic variants reported in ALPS patients. A diagram of the *FAS* protein is shown with the locations of somatic variants. Exons are separated by dashed lines and indicated with grey numbers. The transmembrane and death domains of the protein are highlighted in grey and amino acid numbers are indicated. Details of all variants are shown in **Table 2**.

Identification of somatic *FAS* mutations is becoming more accessible with the advent of new deep sequencing technologies. However, their interpretation remains challenging because of the complex link between genetic variants and their functional effects. We performed a literature search to gather and describe all pathogenic and likely pathogenic somatic *FAS* variants associated with ALPS. We manually inspected all indexed papers describing this type of variation and found 40 variants (including the one described in this study): 18 frameshift, 12 missense, 7 nonsense, 1 in-frame deletion, and 2 splice variants (**Figure 4** and **Table 2**). Most were located in the death domain of exon 9 (15 of 40, 37.5%), including the novel frameshift variant described here, thus supporting the relevance of the *FAS* protein intracellular domain in the development of ALPS.

**Table 2:** Somatic *FAS* variants associated with ALPS.

Exon	Region	Position (hg38)	Nucleotide change	Amino acid change	Reference
2	Extracellular	chr10:89003098	c.100G>T	p.G34X	(25)
3	Extracellular	chr10:89007700	c.197G>A	p.G66D	(26)
3	Extracellular	chr10:89007835	c.332A>G	p.H111R^a^	(17)
5	Extracellular	chr10:89010566	c.471C>A	p.C157X	(26)
6	Transmembrane	n.a.	n.a. (donor splice-site)	p.W189fs (stop at 225)^a^	(15)
6	Transmembrane	n.a.	n.a.	p.W189fs (stop at 220)	(16)
7	Intracellular	chr10:89012061	c.631G>T	p.E211X^a^	(17)
7	Intracellular	n.a.	n.a.	p.P217fs (stop at 220)	(16)
8	Intracellular	chr10:89012083	c.651+2T>C	p.P217fs^a^	(15)
8	Intracellular	chr10:89013368	c.676+1G>A	p.E218MfsX4^a^	(17)
8	Intracellular	chr10:89013334	c.652-9_653del	p.E218MfsX4^a^	(17)
8	Intracellular	chr10:89013342	c.652-1G>T	p.E218MfsX4^a^	(17)
8	Intracellular	chr10:89013367	c.676G>T	p.E218MfsX4^a^	(17)
8	Intracellular	chr10:89013343	c.652G>T	p.E218X^a^	(17)
8	Intracellular	chr10:89013348	c.657_658del	p.V220GfsX6	(17)
8	Intracellular	chr10:89013348	c.657_658delAG	p.V220GfsX6	(27)
8	Intracellular	chr10:89013359	c.668_675del	p.N223RfsX3	(17)
8	Intracellular	chr10:89013362	c.671T>G	p.L224X^a^	(17)
8	Intracellular	chr10:89013365	c.674C>A	p.S225Y^b^	(26)
8	Intracellular	n.a.	n.a.	p.D226fs (stop at 277)^b^	(16)
8	Intracellular	chr10:89013341	c.652-2A>T	Splice	(27)
8	Intracellular	chr10:89013342	c.652-1G>A	Splice	(27)
9	Intracellular	chr10:89014121	c.679delG	p.V227LfsX3	(28)
9	Intracellular	chr10:89014122	c.682_686del	p.D228EfsX2	(17)
9	Intracellular	chr10:89014128	c.686T>A	p.L229X	(29)
9	Intracellular (DD)	n.a.	n.a. (deletion of 8 bp)	p.S230fs (stop at 243)^a^	(15)
9	Intracellular (DD)	n.a.	n.a.	p.S230fs (stop at 240)	(16)
9	Intracellular (DD)	chr10:89014151	c.709G>C	p.A237P	(25)
9	Intracellular (DD)	chr10:89014154	c.712G>T	p.G238X	(27)
9	Intracellular (DD)	chr10:89014161	c.718_719insGTCG	p.M240SfsX8	This study
9	Intracellular (DD)	chr10:89014191	c.749G>A	p.R250Q	(16)
9	Intracellular (DD)	chr10:89014190	c.768C>G	p.R250G	(27)
9	Intracellular (DD)	chr10:89014221	c.779A>T	p.D260V^a^	(15)
9	Intracellular (DD)	chr10:89014220	c.778G>A	p.D260N^a^	(17)
9	Intracellular (DD)	chr10:89014221	c.779A>G	p.D260G	(29)
9	Intracellular (DD)	chr10:89014220	c.778G>T	p.D260Y	(27)
9	Intracellular (DD)	chr10:89014251	c.812_814del	p.A271del	(27)
9	Intracellular (DD)	chr10:89014257	c.815A>G	p.E272G	(27)
9	Intracellular (DD)	chr10:89014259	c.817C>A	p.Q273K	(16)
9	Intracellular (DD)	n.a.	n.a. (1 bp insertion)	p.V275fs (stop at 280)	(25)

DD, death domain; n.a., value not available in the original paper.

a Nomenclature adjusted to the current Human Genome Variation Society (HGVS) guidelines.

b Amino acid adjusted to the canonical transcript (NM_000043.6).

## Discussion

Study of somatic variants underlying inborn errors of immunity (IEI) is gaining increasing interest due to biological, technological, and clinical reasons (30). In 2004, ALPS was the first IEI in which somatic mosaicism was reported as the cause of the disease (15). Since then, other IEIs caused by somatic mutations have been described, with autoinflammatory disorders having the highest incidence (19, 30). Many IEIs resulting from mosaicism are also germline disorders, as in the case of most autoinflammatory disorders and ALPS, whereas few IEIs are predominantly caused by somatic mutations (eg, VEXAS syndrome, TLR8 gain-of-function, and Ras-associated autoimmune leukoproliferative disorder) (31–33).

In this study, we identified a novel somatic mutation in *FAS* by Sanger sequencing of purified CD3^+^ cells and followed the evolutionary dynamics of the variant over time with DAS. Detection of somatic variants at low VAF using classic molecular screening methods such as Sanger is challenging. The variant may go unnoticed or the variant peak on the chromatogram may be mistaken for background noise. In our case, we were fortunate that the variant was a frameshift. The double peak pattern, although the height was small, was highly suspicious for a somatic variant. Moreover, it was specific to CD3^+^ cells and not present in whole blood. However, the single small peak of a single nucleotide variant could be easily misinterpreted as background noise. Hence, we believe that Sanger sequencing on purified CD3^+^ cells may not be sensitive enough for use as a routine diagnostic method in these cases.

NGS-based methods are highly suitable for detecting somatic variants, although their precision and robustness relies on the read depth. Whole-exome sequencing (WES) and whole-genome sequencing (WGS) are intended to identify germline variants, as their mean coverage is usually <200x and <100x, respectively. They can also be used for somatic variant identification, but typically cannot easily detect those at <10% VAF. Targeted gene panels usually have a higher overall read depth (200x-500x). Recently, López-Nevado et al. used a targeted gene panel in whole blood samples to detect germline and somatic *FAS* mutations in ALPS patients (28). These authors identified a pathogenic somatic *FAS* variant in a patient who had no pathogenic germline variants in the panel. To validate the somatic variant call, they used blood DNA from seven other ALPS patients previously diagnosed with somatic *FAS* variants. In all patients but one, they were able to detect somatic variants in a VAF range of 1.9% to 11.5% with a variable read depth in the specific position (101x to 399x). Interestingly, the single patient with a previously identified somatic *FAS* variant that went undetected with this method had DNT counts within the reference range. In the present study, the higher-depth NGS approach (DAS coverage >20,000x) enabled detection of the somatic variant in blood samples containing a very low percentage of DNTs (<1%). The most striking example is detection of the somatic variant at 0.67% VAF in a sample obtained after five years of immunosuppressive treatment. This is a proof of concept that pathogenic somatic *FAS* variants can be detected in whole blood of ALPS patients regardless of their DNT counts with the use of DAS. For that purpose, high coverage of the target gene or exons is needed to ensure identification of variants having a low percentage of supporting reads. In addition, specific bioinformatic processing of the data is required to avoid filtering of true somatic variants by conventional germline-focused strategies. Various somatic variant callers have been created and compared in this regard (20, 34, 35). These are especially useful when the number of candidate genes or exons is not suitable for manual analysis.

One helpful finding obtained here is that a simple CD3^+^ enrichment step may be useful to enhance the sensitivity and reliability of NGS for somatic *FAS* variant detection. The VAF of the somatic variant in our patient was 2.5-fold higher in the CD3^+^ sample than in whole blood, whereas the background noise (artefactual variants) was similar in the two samples. Thus, the probability that true somatic variants would be detected increased after enrichment.

Traditionally, the genetic diagnosis of somatic variants in ALPS has been done by sorting and sequencing DNTs (15, 36). Nonetheless, this is a time- and resource-consuming strategy that may not be available in all centers. López-Nevado et al. demonstrated that somatic *FAS* variants can be detected by NGS-based targeted gene panel analysis in blood of patients with elevated DNTs (28). However, their results showed that the depth of coverage with this approach may not suffice to disclose variants for which only a few reads support the alternative allele (low VAF). We found that NGS-based DAS can detect somatic variants in ALPS patients even when DNTs are within the reference range. In our opinion, a targeted gene panel would be an appropriate initial genetic approach for patients with ALPS suspicion, since it has the advantage of covering germinal mutations in *FAS* (and other ALPS-related genes) and it may detect somatic variants in some cases. Nevertheless, their limited sensitivity may not detect somatic variants at low VAF. Therefore, in case of negative results in a targeted gene panel, DAS should be performed since this technique increases the likelihood of finding the somatic variants, especially at low VAF. In both targeted gene panel analysis and DAS analysis, the PCR steps may introduce sequence errors that confound identification of somatic variants with a very low VAF (<1%-2%). To overcome this limitation in our DAS technique, we applied the quality scores provided by VarScan 2, to identify variants and distinguish true somatic variants from background noise. It is likely that use of CD3^+^-enriched samples and more affordable coverage (5000x) will enable identification of somatic variants in a diagnostic setting. In addition, the method could be useful for patients who have started immunosuppressive treatment without a molecular diagnosis. This opens the door to revisiting previously unsolved cases and may increase the genetic diagnosis rate in pediatric ALPS.

To our knowledge, this is the first reported description of the evolutionary dynamics of a somatic *FAS* mutation in ALPS. We detected the pathogenic somatic variant in all six blood samples collected over a five-year period, including those obtained during sirolimus treatment, which had normal DNT counts. Our results indicate that with a proper approach, genetic assessment of somatic *FAS* variants in ALPS patients can be performed at any time over the clinical course, as immunosuppressive treatment did not eliminate all mutated cells, although their numbers in peripheral blood were considerably reduced. This is consistent with the fact that somatic mutations initially occur in hematopoietic progenitor cells (HPCs) and become enriched in DNTs due to a selective survival advantage (15). HPCs provide a constant supply of mutated descendant cells that are impossible to eliminate with immune suppression (the only known cure is HSCT); thus, the need for chronic treatment in ALPS patients (4, 37). We observed a strong positive correlation between DNT count and the VAF. This finding confirms that somatic *FAS* mutations are mainly restricted to DNTs, although they can be found in varying percentages in other leukocyte subsets (15).

The pathogenic somatic variant reported in this study (c.718_719insGTCG, p.Met240SerfsX8) is located in the intracellular region, within the DD. This domain is essential for formation of the death-inducing signaling complex (DISC), which triggers the extrinsic apoptosis pathway through stimulation by death ligands (38). We reviewed all reported somatic *FAS* mutations and found 40 variants. The majority (34 of 40, 85%) were located in the intracellular domain of the protein and 15 (37.5% of the total) specifically in the DD. Few variants were found in the extracellular or transmembrane domains (4 and 2 respectively, 10% and 5% of the total). The clinical penetrance of intracellular mutations is higher than that of mutations located in the extracellular domain, as intracellular mutant proteins exert a dominant-negative effect, interfering with assembly of *FAS* homotrimers within the cell membrane (39). Interestingly, most of the somatic missense variants reported (9 of 12, 75%) were located in the DD, where missense mutations show highest penetrance (16).

In summary, our study illustrates the evolutionary dynamics of a somatic *FAS* mutation before and during immunosuppressive treatment. We found that the use of high-coverage DAS enabled detection of the somatic variant in whole blood samples regardless of the percentage of DNTs. This opens the possibility to perform genetic testing in patients with suspected somatic ALPS who are receiving treatment. We demonstrated that the VAF strongly correlated with DNT counts and proposed a simple CD3+ enrichment step to increase the probability of detecting true somatic variants in patients with DNTs within the reference range. The capability of the proposed method will facilitate future identification of somatic *FAS* variants and help other researchers and clinicians on their way toward a genetic diagnosis.

## Data availability statement

The data presented in the study are deposited in the ClinVar repository. This data can be found here: Link: https://www.ncbi.nlm.nih.gov/clinvar/variation/1703254/?oq=SCV002568438&m=NM_000043.6(FAS):c.718_719insGTCG%20(p.Met240fs) / accession number: SCV002568438.

## Ethics statement

The studies involving human participants were reviewed and approved by Ethics Review Board of Hospital Universitari Vall d’Hebron [code: PR(AG)69/2016]. Written informed consent to participate in this study was provided by the participants’ legal guardian/next of kin. Written informed consent was obtained from the minor(s)’ legal guardian/next of kin for the publication of any potentially identifiable images or data included in this article.

## Author contributions

LB-M performed the bioinformatics analysis of NGS data, somatic variant calling, and the literature review. MG-P, AP-M, and CF-J performed laboratory analyses and CD3^+^ purification. MM-G performed flow cytometry studies. AA-C performed sample collection and Sanger sequencing. PV, JR, AM-N, and PS-P provided patient care, and collected and provided clinical data. MA was responsible for the NGS technical process, including library preparation and sequencing. LB-M and RC analyzed and interpreted the data, and wrote the manuscript. RC designed and supervised the project, provided resources, and edited the manuscript. All co-authors reviewed, commented on, and approved the final version of the manuscript.

## Funding

This study was funded by Instituto de Salud Carlos III, grant PI20/00761, cofinanced by the European Regional Development Fund (ERDF).

## Acknowledgments

This research is supported by the European Reference Network for Rare Immunodeficiency, Autoinflammatory and Autoimmune Diseases (ERN-RITA).

## Conflict of interest

The authors declare that the research was conducted in the absence of any commercial or financial relationships that could be construed as a potential conflict of interest.

## Publisher’s note

All claims expressed in this article are solely those of the authors and do not necessarily represent those of their affiliated organizations, or those of the publisher, the editors and the reviewers. Any product that may be evaluated in this article, or claim that may be made by its manufacturer, is not guaranteed or endorsed by the publisher.
