# Supplementary material for: Detection and evolutionary dynamics of somatic FAS variants in autoimmune lymphoproliferative syndrome: Diagnostic implications
Source: Front Immunol. 2022 Nov 18;13:1014984. doi: 10.3389/fimmu.2022.1014984 (PMC9716137; doi:10.3389/fimmu.2022.1014984)
Supplement: Supplementary file 1 [file DataSheet_1.pdf]

Detection and evolutionary dynamics of somatic FAS variants in autoimmune lymphoproliferative syndrome: diagnostic implications

Laura Batlle-Masó, Marina Garcia-Prat, Alba Parra-Martínez, Clara Franco-Jarava, Aina Aguiló-Cucurull, Pablo Velasco, María Antolín, Jacques G. Rivière, Andrea Martín-Nalda, Pere Soler-Palacín, Mónica Martínez-Gallo, Roger Colobran

Supplementary information

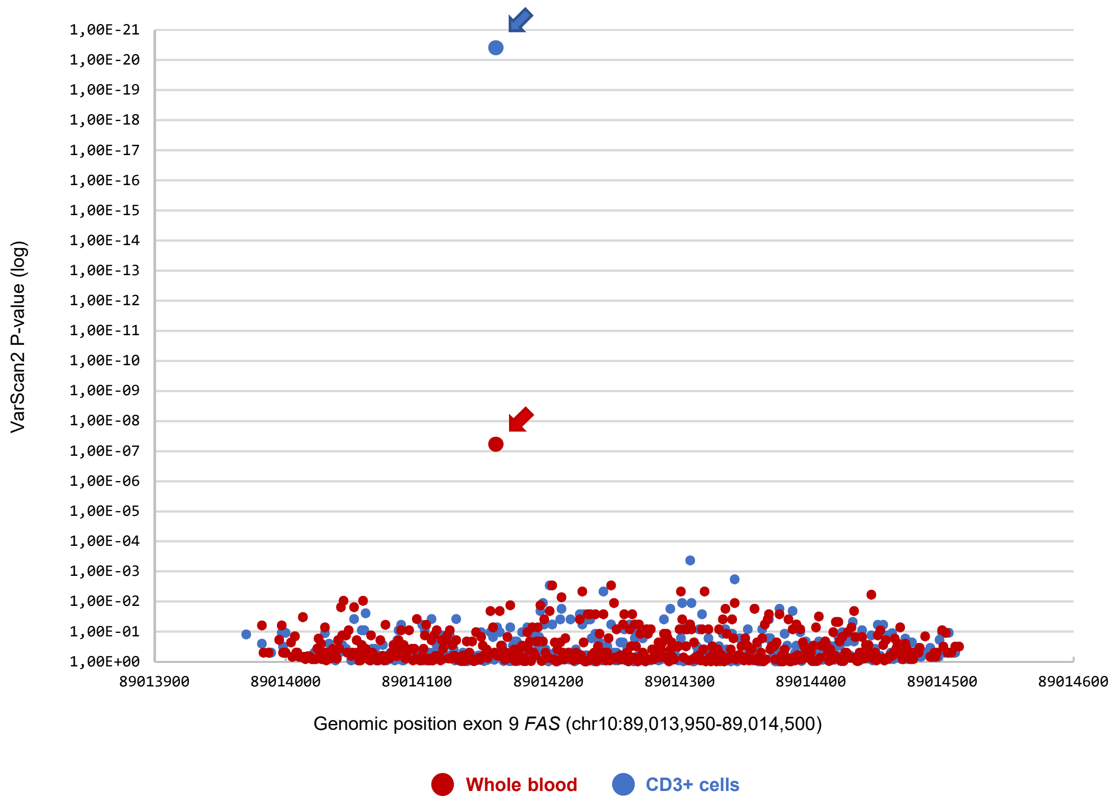

Supplementary Figure 1. All genetic variants called by VarScan2 in exon 9 of the FAS gene (hg38) versus their p value are shown. P values given by VarScan2 are represented in inversed order and using logarithmic values. The pathogenic somatic variant (chr10:89,014,160, c.718_719insGTCG) is indicated with an arrow
.
