# Supplementary material for: Detection and evolutionary dynamics of somatic FAS variants in autoimmune lymphoproliferative syndrome: Diagnostic implications
Source: Front Immunol. 2022 Nov 18;13:1014984. doi: 10.3389/fimmu.2022.1014984 (PMC9716137; doi:10.3389/fimmu.2022.1014984)
Supplement: Supplementary file 2 [file DataSheet_2.pdf]

Detection and evolutionary dynamics of somatic FAS variants in autoimmune lymphoproliferative syndrome: diagnostic implications

Laura Batlle-Masó, Marina Garcia-Prat, Alba Parra-Martínez, Clara Franco-Jarava, Aina Aguiló-Cucurull, Pablo Velasco, María Antolín, Jacques G. Rivière, Andrea Martín-Nalda, Pere Soler-Palacín, Mónica Martínez-Gallo, Roger Colobran

Supplementary Table 1. Primers and PCR conditions for *FAS*, *CASP8*, and *CASP10* amplification and subsequent sequencing by Sanger method. PCR conditions (35 cycles) were as follows: 95°C for 30 s, annealing for 30 s (see specific temperatures in the table) and 72°C for 45 s.

Exon[a]	Primer forward (5'-3')	Primer reverse (5'-3')	Amplimer	Tª annealing
FAS				
1	AAGCCTTTAGAAAGGGCAGGAGG	GGCCTGAGGGAAGGTCCTAACAG	707 bp	65°C
2	CAGTGGAGCCCTCACATTGTC	TGGAGCCAAATAGACCAGTCTTCTC	553 bp	66°C
3	ATCTCAGTGGCCAGTAACA	TATCTGGAAGAATTGCCTAGAC	735 bp	66°C
4	CTGGTGTCATGCTGTGACTGTTG	TAGTTGCTTGGAGGCAAAGCAGG	364 bp	66°C
5-6	ATACGTTTGCCAGAGATGCAAAG	AAGAATGAGGCAAATCTTTGTGAAC	471 bp	64°C
7	GGCCAGCCTGTGGTAATTAGATG	CACTTGAGCCCAGGACTTTGAG	515 bp	66°C

8	GCTTAGTTTCTGGCAAGGCCG	GAAAAGTTTCTTAAGCCTCAGACTATTG	481 bp	64°C
9	TCTTGTTTCTGTATTCCCCTAGTCAGC	AAACCAAGCAGTATTTACAGCCAGC	570 bp	62°C
CASP8				
1	CATTGGCTTGTTTGTATGTCTA	CCACCCAAGGCACTACTG	673 bp	64°C
2	TCATAAACCATGCCATTAACTG	GAGCAGCACTTCGGTCTAAA	417 bp	64°C
3-4	ACATGGCTTCTCCTTTATCCTC	GTAGAGCAGGCACGTTAAGATG	640 bp	66°C
5	CCCCTGTTCCCAGCTTGAAT	GCTGGCACGGCTGCTAGA	497 bp	66°C
6	GAAACGACCCCGAGTTGG	CATGGCCCTTTTGGTATAGCTT	362 bp	66°C
7	GAGAGGGAGAGGGGTCAAATTC	GGGGGAAGGTAGTGTAACAGTG	662 bp	66°C
8	GTCTTGGTTTCGCACCTTATTG	GGCGACAGAGCGAGATT	342 bp	64°C
CASP10				
1	AATGCCCAGAACGGAGAA	GAGGTGGGCTTCACTGCTAGT	721 bp	66°C
2	GGCCTGCTTCTACCACTTACTG	TGGCCTCATGCTAATGCTT	630 bp	66°C
3	TGTGACCTTCGGCAAGTTAC	AACATAGTGAAACCCTGTCGCT	778 bp	66°C

4	ACTGGCTTTATTTAAGGACCAC	GCCCTGTGCTCCATAGATTAT	701 bp	64°C
5	CTTTTCTGTGGCTGTGGATTAG	GCCTGCAGTCCCTAGAATCT	404 bp	66°C
6	GGCCTTGAGAGAAGCATAATTC	CGTGGTACATCTTTATCCAAGC	542 bp	64°C
7	ATCACAAGGTCAAGAGATCAAG	AAGAGGCTCAGAAACATGATA	780 bp	60°C
8	AGTTCTTCATTTTGTTTTGCAT	AACGTAGAACTCTCACCCTTAC	679 bp	60°C
9	GGCCTCTCCACTTGAGTTATCA	TGTCAATCTCAGGCGATGT	487 bp	66°C

[a]Only coding exons have been sequenced.
